# The Potential Role of Angiotensin-Converting Enzyme Inhibitors and Beta-Blockers in Reducing Pneumonia Severity in Older Adults

**DOI:** 10.7759/cureus.57463

**Published:** 2024-04-02

**Authors:** Heledd Thomas, Yuki Yoshimatsu, Trevor Thompson, David G Smithard

**Affiliations:** 1 Elderly Care, Queen Elizabeth Hospital, Lewisham and Greenwich NHS Trust, London, GBR; 2 Centre for Chronic Illness and Ageing, University of Greenwich, London, GBR

**Keywords:** angiotensin-converting enzyme inhibitors, beta-blockers, community-acquired pneumonia, curb-65 score, pneumonia severity index, aspiration pneumonia

## Abstract

Background

Understanding the impact of pharmacological therapy on pneumonia severity is crucial for effective clinical management. The impact of angiotensin-converting enzyme inhibitors (ACEis) and beta-blockers (BBs) on pneumonia severity remains unknown, warranting further investigation.

Methodology

This retrospective study examined the hospital records of inpatients (≥75 years) admitted with community-acquired pneumonia in 2021. Pneumonia severity associated with the use of pre-established ACEi and BB therapy was documented using CURB-65 (confusion, uraemia, respiratory rate, blood pressure, age ≥65 years) and pneumonia severity index (PSI) scores. Descriptive statistics and multivariable linear regression were used to analyse differences across BB therapy, ACEi therapy, their combination, or neither (control group).

Results

A total of 803 patient records were examined, of whom 382 (47.6%) were male and 421 (52.4%) were female. Sample sizes for each group were as follows: control (n = 492), BB only (n = 185), ACEi only (n = 68), and BB + ACEi (n = 58). Distribution of aspiration pneumonia (AP) versus non-AP for each group, respectively, was control (21.1% vs. 78.9%), BB only (9.7% vs. 90.3%), ACEi only (7.3% vs. 92.7%), and ACEi + BB (12.1% vs. 87.9%). No significant differences in PSI and CURB-65 scores were found between intervention groups even after controlling for patient characteristics and irrespective of AP or non-AP aetiology. Patients with AP had significantly higher CURB-65 (p = 0.026) and PSI scores (p = 0.044) compared to those with non-AP.

Conclusions

Pre-prescribed ACEi or BB therapy did not appear to be associated with differences in pneumonia severity. There were no differences in pneumonia severity scores with ACEi and BB monotherapy or combined ACEi and BB therapy.

## Introduction

Pneumonia is a leading cause of morbidity and mortality among older adults worldwide [1-5]. The burden of pneumonia-associated hospitalisation of older adults is significant, particularly in individuals with underlying comorbidities such as cardiac failure, dementia, neurological disorders, diabetes, and chronic lung disease, making them more susceptible to pneumonia [4-8]. Hence, there is increasing interest in exploring the potential role of angiotensin-converting enzyme inhibitors (ACEis) and beta-blockers (BBs), drugs used for chronic cardiovascular disease, in reducing pneumonia severity [3,9].

ACEis, such as perindopril, have been shown to reduce the risk of pneumonia in patients with a history of stroke or transient ischaemic attack [3,10]. Their protective effect may be mediated by their anti-inflammatory effects on the pulmonary system [11-14]. ACEis inhibit the activity of angiotensin-converting enzyme (ACE), preventing the conversion of the peptide hormone angiotensin I into angiotensin II. Angiotensin II, a potent vasoconstrictor, has been shown to promote lung oedema, inflammation, impaired lung function, and lung injury in pneumonia [11,13]. This may be the basis for ACEi and angiotensin receptor blocker (ARB) effects in reducing the risk of pneumonia and improving pneumonia-related outcomes [14-17].

The use of ACEis has been found to have a protective effect against the recurrence of aspiration pneumonia (AP) [18]. Current evidence suggests ACEis have adverse pleiotropic effects on the respiratory system, such as stimulating the cough reflex [19]. This adverse effect is thought to be related to the accumulation of the pro-inflammatory neuropeptide, substance P in the upper respiratory tract due to the blockade of ACE activity [3]. Substance P sensitises the sensory nerves of the airways and enhances the cough reflex, and this increased cough reflex may protect the tracheobronchial tree, improving swallowing by reducing exposure of the respiratory tree to oropharyngeal secretions [3,20,21]. Elevated levels of substance P, associated with an ACEi-induced cough, may also contribute to a decreased risk of AP among ACEi-treated patients [3]. Additionally, ACEis increase levels of the neuropeptide, bradykinin by inhibiting its degradation. Similar to that of substance P, bradykinin sensitises the cough reflex, thereby potentially contributing to decreasing the risk of AP among ACEi-treated patients [21]. While some studies have suggested a decreased risk of pneumonia with ACEis [3,15,16,22,23], others have found conflicting evidence, highlighting the need for further study [24,25].

BBs are thought to improve endothelial function and reduce tachycardia in respiratory infections [26-30]. They are also known to inhibit the release of pro-inflammatory cytokines, interleukin-6 (IL-6), tumour necrosis factor-alpha (TNF-α), and interleukin-1β (IL-1β), reducing pneumonia severity and mortality [31,32]. Similarly, to that of ACEis, BBs have been found to reduce the risk of pneumonia in post-stroke patients [33]. BBs have been shown to protect against oropharyngeal dysphagia by increasing the level of substance P in saliva, which could contribute to protection against AP [34]. However, the specific mechanisms by which BBs exert their anti-inflammatory effects in pneumonia are not fully understood and require further investigation.

Despite this interest, limited empirical studies have explored the potential benefits of ACEis and BBs therapy in reducing pneumonia severity and improving outcomes. The impact of BBs on pneumonia outcomes is less well-studied compared to that of ACEis. It is important to note that the effects of these drugs on pneumonia outcomes may vary depending on factors such as ethnicity, age, sex, and comorbidities [10,23]. Therefore, we evaluate the efficacy of ACEi monotherapy, BB monotherapy, and their combination in relation to no treatment, while examining potential influences on treatment efficacy by factors such as age, sex, and comorbidities. Hence, this study hypothesises that older adults pre-established on ACEis, BBs, or a combination of both may suffer pneumonia of reduced severity compared with those taking neither ACEis nor BBs.

This paper was previously presented at the 19th European Geriatric Medicine Society Conference in September 2023.

## Materials and methods

Study design

A retrospective, cross-sectional study was conducted to compare pneumonia severity scores, specifically the Pneumonia Severity Index (PSI) and CURB-65 (confusion, uraemia, respiratory rate, blood pressure, age ≥65 years), across the following four treatment groups: individuals pre-established on BB monotherapy, ACEi monotherapy, combination BB and ACEi therapy, or neither BB nor ACEi (control group). The primary objective was to identify differences in pneumonia severity scores among the four specified treatment groups. The study also compared PSI and CURB-65 scores among individuals diagnosed with AP versus those diagnosed with non-AP. The secondary objective was to identify any differences in the severity of AP compared to non-AP within the entire cohort. Study data was collated using electronic patient record data from Queen Elizabeth Hospital, a district general hospital in South East London under Lewisham and Greenwich NHS Trust.

Ethical considerations

This study was approved by Lewisham and Greenwich NHS Trust as instituted by the Declaration of Helsinki (Number 7211). The use of anonymised, retrospective data meant individual patient consent was waived.

Data selection and inclusion criteria

A total of 803 patients were included, who were 75 years or older, admitted as inpatients with a diagnosis of non-aspiration or aspiration community-acquired pneumonia (CAP) between January 1, 2021, and December 31, 2021. A list of patients diagnosed with pneumonia as their primary or secondary diagnosis was compiled from the hospital database. CAP was characterised as pneumonia developing in the community or within 48 hours of hospital admission. The diagnosis was established through analysis of documentation from the admitting consultant physician’s ward round at the time of admission. Non-AP was defined as any case of CAP with no documentation indicating aspiration as its aetiological factor, such as documented dysphagia or recent hospital admission. Patients with both CAP and infectious exacerbation of chronic obstructive pulmonary disease were also included. Patients admitted more than once during the study duration were considered only for their first admission, with subsequent admissions excluded from the analysis. Exclusion criteria included patients diagnosed with hospital-acquired pneumonia (defined as a pneumonia diagnosis >48 hours after admission) or COVID-19 pneumonitis, those admitted for a different condition or cases where medical record documentation of diagnosis was unclear (coded data differed from written documentation). Patient data including age, sex, median Rockwood clinical frailty scale (CFS) score, pneumonia history within one year, and comorbidities (including cardiac ischaemic/congestive disease, stroke, neurological disorders, dementia, mental disorders, respiratory disorders and diabetes mellitus (DM)) were collated from electronic medical records and recorded in an Excel spreadsheet. CURB-65 and PSI scores were calculated for each patient using admission medical record data.

Statistical analysis

To examine potential differences in patient characteristics across the four treatment groups, chi-square tests (categorical characteristics) or one-way analysis of variance (continuous characteristics) were conducted examining age, median Rockwood CFS score, sex, cardiac ischaemic/congestive disease, stroke, neurological disorder, dementia, mental disorder, respiratory disorder, DM, and pneumonia history within one year.

Linear regression was conducted with dummy-coded predictors of intervention (BB, ACEi, BB + ACEi vs. the no intervention reference - control group) and pneumonia type (AP vs. non-AP) with outcomes of PSI and CURB-65 scores. Adjusted analysis was then performed by repeating the regression while controlling for patient characteristics of age, sex, Rockwood CFS score, comorbidities, and pneumonia history within one year.

## Results

Patient demographics

A total of 803 patient records were examined, with 382 (47.6%) being male and 421 (52.4%) being female. In total, 134 patients were diagnosed with AP and 669 were diagnosed with non-AP (n = 669). The sample sizes for each treatment group were as follows: control (n = 492), BB only (n = 185), ACEi only (n = 68), and BB + ACEi (n = 58). The percentage of patients diagnosed with AP versus non-AP within each treatment group is shown inTable 1.

**Table 1:** Distribution of AP versus non-AP within each treatment group. AP = aspiration pneumonia; BB = beta-blockers; ACEi = angiotensin-converting enzyme inhibitors

Treatment group	AP (n = 134)		Non-AP (n = 669)	
	n	%	n	%
Control (n = 492)	104	21.1	388	78.9
BB only (n = 185)	18	9.7	167	90.3
ACEi only (n = 68)	5	7.3	63	92.7
ACEi + BB (n = 58)	7	12.1	51	87.9

The control group had the highest proportion of patients diagnosed with AP (21.1%), followed by the ACEi + BB group (12.1%), the BB-only group (9.7%), and the ACEi-only group (7.3%). The majority of patients in each group were diagnosed with non-AP rather than AP, with proportions of non-AP ranging from lowest, 78.9%, in the control group to highest, 92.7%, in the ACEi-only group.

Patient characteristics across treatment groups

As shown in Table 2, there were no significant differences across treatment groups on baseline characteristics of stroke, respiratory disorders, pneumonia history, or male sex. However, there were significant differences between treatment groups in age (p = 0.012), proportion of patients with cardiac ischaemic/congestive disease (p < 0.001), dementia (p = 0.005), neurological conditions (p = 0.008), mental health disorder (p = 0.033), and DM (p = 0.002) compared to the control group.

**Table 2:** Differences in patient characteristics across treatment groups. Asterisks (*) denote statistical significance levels as follows: * p < 0.05, ** p < 0.01, *** p < 0.001. BB = beta-blockers; ACEi = angiotensin-converting enzyme inhibitors; CFS = clinical frailty severity; DM = diabetes mellitus

Patient characteristics	Control (%) (n = 492)	ACEi only (%) (n = 68)	BB + ACEi (%) (n = 58)	BB only (%) (n = 185)	P-value
Age on admission (mean)	85.15	83.87	82.52	84.82	0.012^*^
CFS Rockwood score (median)	5	4	5	5	<0.001^***^
Percentage with characteristics					
Male sex	54	43	50	52	0.350
Cardiac ischaemic/congestive disease	18	31	52	54	<0.001^***^
Stroke	15	18	18	17	0.813
Neurological disorder	8	0	2	4	0.008^**^
Dementia	32	24	12	24	0.005^**^
Mental disorder	12	4	16	6	0.033^*^
Respiratory disorder	25	24	28	34	0.110
DM	18	34	29	29	0.002^**^
Pneumonia history within one year	22	16	22	22	0.759

**Figure 1:**
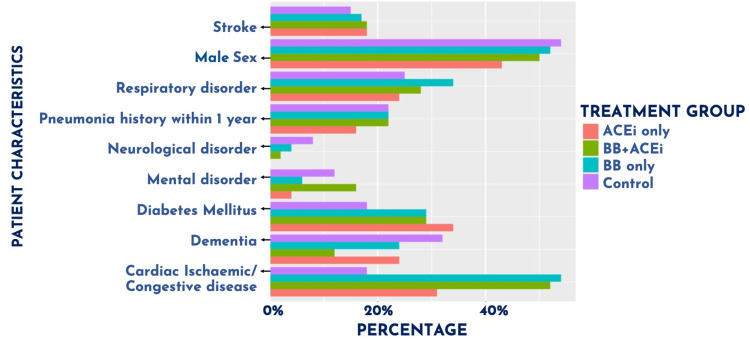
Differences in patient characteristics across treatment groups. The graph illustrates the differences in the percentage distribution of patient characteristics, including age, sex, pneumonia history within one year, and comorbidities within each intervention group - BB monotherapy, ACEi monotherapy, BB + ACEi therapy, and control group (neither BB or ACEi therapy). BB = beta-blockers; ACEi = angiotensin-converting enzyme inhibitors

Specifically, post-hoc comparisons showed that, compared to the control group, all intervention groups exhibited significantly higher proportions of cases of cardiac ischaemic/congestive disease (p = 0.013-0.001) and diabetes mellitus (p = 0.005-0.001), as shown in Figure 1 and Table 2.

Additionally, the BB and BB + ACEi combined therapy groups showed a higher proportion of dementia (p = 0.002-0.001), BB monotherapy exhibited a higher proportion of mental health disorders (p = 0.001), and the BB + ACEi combined therapy group showed a higher proportion of neurological conditions (p = 0.015-0.001). Post hoc t-tests also showed the BB monotherapy group to be significantly lower in age than the control group, although the magnitude of this difference was small (M = 84.8 vs, 85.2 years, respectively).

Comparison of Pneumonia Severity Index and CURB-65 scores among treatment groups

After controlling for patient characteristic confounders, no significant differences were found in the PSI and CURB-65 scores among all four treatment groups, as shown in Table 3. However, PSI and CURB-65 severity scores were significantly higher in patients diagnosed with AP compared to non-AP across all four groups (p = 0.026, p = 0.044, respectively) (Table 3).

**Table 3:** Differences in Pneumonia Severity Index (PSI) and CURB-65 scores among treatment groups. Asterisks (*) denote statistical significance levels as follows: * p < 0.05, ** p < 0.01, *** p < 0.001. PSI = Pneumonia Severity Index; CURB-65 = confusion, uraemia, respiratory rate, blood pressure, age ≥65 years; BB = beta-blockers; ACEi = angiotensin-converting enzyme inhibitors; AP = aspiration pneumonia; EOM = estimate of mean; SEM = standard error of mean; DM = diabetes mellitus; CFS = clinical frailty scale

Treatment group	CURB-65				PSI			
	EOM	Conf.low	Conf.high	p-value	EOM	Conf. low	Conf. high	P-value
BB only (vs. control)	0.06	-0.09	0.20	0.459	1.64	-2.29	5.58	0.412
ACEi only (vs. control)	-0.05	-0.26	0.16	0.643	-2.27	-7.93	3.40	0.432
ACEi + BB (vs. control)	0.17	-0.05	0.40	0.136	5.29	-0.84	11.40	0.091
AP diagnosis	0.17	0.00	0.33	0.044^*^	4.88	0.58	9.19	0.026^*^
Age on admission	0.01	0.00	0.02	0.060	1.04	0.78	1.30	<0.001^***^
Male sex	-0.09	-0.20	0.03	0.135	-12.10	-15.20	-8.97	<0.001^***^
Cardiac ischaemic/Congestive disease	-0.05	-0.18	0.08	0.464	0.95	-2.61	4.51	0.600
Stroke	0.10	-0.07	0.26	0.241	3.88	-0.45	8.21	0.079
Neurological disorder	0.15	-0.09	0.39	0.231	1.60	-4.88	8.07	0.628
Dementia	0.35	0.21	0.49	<0.001^***^	2.45	-1.33	6.24	0.203
Mental disorder	-0.05	-0.24	0.14	0.597	-2.60	-7.62	2.42	0.309
Respiratory disorder	0.02	-0.12	0.15	0.816	0.89	-2.63	4.41	0.620
DM	0.05	-0.09	0.18	0.517	0.34	-3.35	4.02	0.858
Pneumonia history within one year	0.02	-0.12	0.16	0.767	0.56	-3.25	4.37	0.773
CFS Rockwood score	0.08	0.04	0.12	<0.001^***^	2.36	1.22	3.50	<0.001^***^

## Discussion

This study aimed to investigate the relationship between pre-prescribed ACEi monotherapy, BB monotherapy, BB + ACEi combined therapy, or neither drug with the severity of pneumonia in patients aged 75 and above admitted with CAP in 2021. The primary end-point was measured using pneumonia severity scoring systems, CURB-65 and PSI. Study outcomes provide valuable insights into the relationship between ACEi monotherapy, BB monotherapy, BB + ACEi combined therapy, and neither drug, specifically addressing AP risk, along with individual patient characteristics that may influence the severity of the disease.

After controlling for patient characteristics, no significant differences were found in the PSI and CURB-65 scores across treatment groups suggesting that the medication regimen had no association with disease severity in this study. In congruence with these findings, published studies demonstrate no correlation between ACEi therapy and reduction in pneumonia risk and risk of hospitalisation secondary to CAP [23,35]. Similar findings have been documented with BB therapy [36]. Despite the established anti-inflammatory and pulmonary protective effects of BBs and ACEis, such beneficial effects did not appear to influence pneumonia severity within this study. This may be attributed to factors such as the observational nature of the study, drug dosages, variations in specific drugs within their respective classes, patient compliance, or potential interaction with other prescribed medications. Further investigation in the form of a randomised controlled trial may be beneficial in the future.

Notably, the proportion of patients diagnosed with AP varied significantly across individual treatment groups. The control group exhibited the highest proportion of AP cases (21.1%), while the ACEi + BB group had the lowest proportion (12.1%). These findings suggest that the control group, representing individuals receiving neither ACEi nor BB therapy, may be more predisposed to develop AP, as supported by related studies [37,38]. Further, patients diagnosed with AP rather than non-AP were found to have significantly higher PSI and CURB-65 scores across all four treatment groups, emphasising greater disease severity with AP compared to non-AP, irrespective of the treatment regimen. This is consistent with current literature that reports AP is associated with greater disease severity, more comorbidities, and higher mortality compared to non-AP [39]. Furthermore, the CURB-65 scoring system has been shown to have limited validity in predicting pneumonia mortality in patients with AP, which could lead to inaccuracies in the results of this study [39].

Patient characteristics

Analysis of comorbidities among the treatment groups revealed that patient characteristics play a role in predicting pneumonia severity. Significant differences in the proportion of patients with cardiac ischaemic/congestive disease were observed among the groups. Both the BB + ACEi and BB-only groups exhibited the highest proportions, which may have reduced the beneficial impact of pre-existing treatment on pneumonia severity, given there is an established link between cardiac ischaemic/congestive disease and pneumonia risk. Previous studies have found a reduced risk of pneumonia in patients with a history of stroke or transient ischaemic attack treated with ACEi, supporting this hypothesis [10].

Moreover, the apparent equivocal risk of pneumonia between patients established on ACEi and BB therapy and those not on these regimens could be influenced by the plausible beneficial impacts of these medications on cardiovascular health [40,41]. Research investigating the causes of death among patients with radiological evidence of pneumonia shows that cardiac conditions are the second most frequent cause of mortality, following respiratory failure in this population [42]. ACEis and BBs are routinely prescribed for the management of cardiovascular conditions. Consequently, individuals on these treatments could potentially experience more effective control of their underlying cardiovascular disease, which could conceivably temper the severity of pneumonia or mitigate the frequency of pneumonia-associated exacerbations. This would coincide with previous studies that found the use of ACEis for hypertension, as opposed to other antihypertensive drugs, reduces pneumonia risk, particularly in patients with previous strokes, suggesting beneficial pneumonia prevention effects of ACEis [43]. Similarly, BB therapy has been shown to reduce the risk of pneumonia and frequency of pneumonia in patients with a prior history of ischaemic stroke [33,44]. Furthermore, studies that found a protective effect of ACEis with the development of pneumonia included patients with a history of stroke/cardiovascular disease [45,46].

Additionally, the study also revealed significant differences in the proportion of patients with neurological disorders and dementia among the treatment groups. Notably, the control group exhibited the highest proportion of patients with both conditions. These findings suggest a potential association between neurological disorders, dementia, and AP risk, supporting existing evidence [6]. Given the higher occurrence of aspiration pneumonia in dysphagia and impaired cough reflex, the higher proportion of patients with neurological disorders and dementia in the control group could be attributed to the reduced cough reflex potentially induced by ACEis [47].

Strengths and limitations

The current study has several strengths, including a relatively large sample size and an equitable distribution of sex. Additionally, both ACEis and BBs are prescribed for indications other than pneumonia. This allows for the evaluation of treatment differences without the potential bias of "confounding by indication.” Specifically, eliminating the potential bias that patients with more severe pneumonia are preferentially administered a specific drug compared to patients with less severe pneumonia. Nevertheless, study limitations should be acknowledged. The retrospective design of the study inherently introduces potential biases and constrains the ability to draw causal inferences. Furthermore, the study exclusively focuses on ACEi and BB therapies, neglecting the potential influence of other prescribed medications and potential drug interactions on pneumonia risk. Moreover, the study refrained from investigating details such as the duration of ACEi/BB prescription, the dosage of these medications, or any potential discrepancies between specific ACEi/BB prescriptions, all of which could yield pertinent insights into the temporal relationship with pneumonia severity, given the variable pharmacokinetic effects. For example, studies report that catecholamines predominantly modulate immune cells via beta-2 receptors, which could indicate that beta-1 receptor-selective BBs may exert a more pronounced impact on pneumonia severity compared to beta-2 receptor antagonists [48].

In the context of frailty, there is evidence to suggest that the immune system exhibits heightened activity before infection, followed by an exaggerated response, referred to as “inflammageing” during acute infection. This immune phenomenon may be related to the potential benefits of BB in potentially mitigating this exaggerated immune response. Additionally, the study omitted considerations of ethnicity, a key factor known to influence pneumonia severity [18].

Other limitations of this study are confined to the use of CURB-65 and PSI scoring systems in predicting pneumonia severity. The CURB-65 pneumonia severity scoring system includes parameters such as blood urea nitrogen (BUN) and confusion which in elderly patients could be influenced by other factors unrelated to the severity of respiratory disease [49,50]. For example, confusion in elderly patients can be caused by delirium, medication side effects, or underlying neurological conditions. Similarly, elevated BUN levels can be seen in patients with renal impairment or dehydration. Therefore, when interpreting the CURB-65 score, it is crucial to consider these potential confounding factors. Additionally, studies have found that CURB-65 may not identify patients requiring intensive care admission, implying that the use of the CURB-65 scoring system is a poor predictor of disease severity [49]. The PSI score has a large emphasis on age, classifying patients into different risk classes (1-5, low to very high risk) based on various factors, including patient characteristics, comorbidities, and clinical signs and symptoms. For example, patients over 50 years of age are classified as risk class 2 in terms of pneumonia severity. Consequently, the PSI scoring system may overestimate the severity of pneumonia in elderly but otherwise healthy patients. Despite the limitations of CURB-65 and PSI in the elderly population, it remains the gold standard scoring tool in determining the severity of pneumonia in this population.

## Conclusions

In summary, the findings of this study contribute to our understanding of the interplay between distinct pre-existing medication regimens and the severity of CAP in elderly patients. The results suggest that ACEi, BB, or combination therapies may not affect pneumonia severity in older adults; instead, attributes such as age, comorbidities, and other patient-specific factors emerge as pivotal determinants of outcomes. AP is shown to be associated with an overall higher severity of pneumonia than non-AP which means targeting the prevention of comorbidities and frailty leading to dysphagia to prevent AP is integral. Furthermore, using the established clinical scores to quantify pneumonia severity in older adults introduces complications which make interpretation of findings more difficult. Future prospective investigations encompassing larger cohorts and a more exhaustive prospective assessment of medication utilisation are essential to validate these findings.
